# The impact of kaolin mining activities on bacterial diversity and community structure in the rhizosphere soil of three local plants

**DOI:** 10.3389/fmicb.2024.1424687

**Published:** 2024-09-09

**Authors:** Wei Gao, Xiaodie Chen, Jing He, Ajia Sha, Yuanhang Ren, Peng Wu, Qiang Li

**Affiliations:** ^1^Clinical Medical College and Affiliated Hospital of Chengdu University, Key Laboratory of Coarse Cereal Processing, Ministry of Agriculture and Rural Affairs, Chengdu University, Chengdu, Sichuan, China; ^2^Yunnan Plateau Characteristic Agricultural Industry Research Institute, Yunnan Agricultural University, Kunming, Yunnan, China

**Keywords:** mining activity, soil pollution, rhizosphere bacteria, pollution control, soil

## Abstract

**Introduction:**

Thus far, the impact of kaolin mining activities on the surrounding native plants and rhizosphere microecology has not been fully understood.

**Methods:**

In this study, we used 16S rRNA high-throughput sequencing to examine the impact of kaolin mining on the rhizosphere bacterial communities and functions of three local plant species: *Conyza bonariensis*, *Artemisia annua*, and *Dodonaea viscosa*.

**Results:**

The results showed that kaolin mining significantly reduced the diversity of rhizosphere bacteria in these plants, as indicated by the Shannon, Simpson, Chao1, and observed species indices (*p* < 0.05). Kaolin mining had an impact on the recruitment of three rhizosphere bacteria native to the area: *Actinoplanes*, *RB41*, and *Mycobacterium*. These bacteria were found to be more abundant in the rhizosphere soil of three local plants than in bulk soil, yet the mining of kaolin caused a decrease in their abundance (*p* < 0.05). Interestingly, *Ralstonia* was enriched in the rhizosphere of these plants found in kaolin mining areas, suggesting its resilience to environmental stress. Furthermore, the three plants had different dominant rhizosphere bacterial populations in kaolin mining areas, such as *Nocardioides*, *Pseudarthrobacter*, and *Sphingomonas*, likely due to the unique microecology of the plant rhizosphere. Kaolin mining activities also caused a shift in the functional diversity of rhizosphere bacteria in the three local plants, with each plant displaying different functions to cope with kaolin mining-induced stress, such as increased abundance of the GlpM family and glucan-binding domain.

**Discussion:**

This study is the first to investigate the effects of kaolin mining on the rhizosphere microecology of local plants, thus contributing to the establishment of soil microecological health monitoring indicators to better control soil pollution in kaolin mining areas.

## Introduction

Kaolin is a non-metallic mineral that is a kind of clay and clay rock mainly composed of kaolinite group clay minerals (Kumar and Lingfa, 2020; Hu et al., 2023). Kaolin has excellent physical and chemical properties, such as malleability and fire resistance, and is widely used in papermaking, ceramics, and refractory materials (Menezes et al., 2009; Burt et al., 2021; Miranda et al., 2021). In addition, kaolin has various uses and is widely used in fields such as medicine, agriculture, environmental science, and materials (Menezes et al., 2008; Rammak et al., 2021). Kaolin is mainly used in medicine as a hemostatic material, antibacterial agent (Ricardo and Lipner, 2021; Tamer et al., 2021). In agriculture, it is mainly used as a plant protective agent, which can regulate plant growth and development, quality, stress resistance, disease, and pest resistance (Pazos et al., 2006; Brito et al., 2018; Oliveira et al., 2022; Sang et al., 2022). In the environment, it is mainly used for the adsorption of soil and water pollutants (Moumen et al., 2022). With the increasing demand for kaolin, the scale of kaolin mining is also expanding daily. The extraction and processing of kaolin may have negative environmental effects, such as the diffusion of heavy metals, sewage, and other harmful gases, ultimately affecting the quality and safety of the surrounding groundwater, soil, and air (Osmanlioglu, 2002; Wei et al., 2002; Wang et al., 2006; Dědková et al., 2015). Previous studies have found that the mining of kaolin also brings a series of environmental effects, such as the diffusion of radioactive elements (Adagunodo et al., 2018; Yong et al., 2023). However, the impact of kaolin mining on surrounding plants and microorganisms is still unknown.

Soil rhizosphere microorganisms are one of the important indicators of the ecological health of healthy soil (Xiang et al., 2023; Li L. et al., 2024). Rhizosphere microorganisms play an important ecological function in the process of long-term coexistence with plants (Pronk et al., 2022; Zhang et al., 2023). Rhizosphere bacteria can promote the absorption of elements and nutrients by plants, secrete plant hormones, and enhance the tolerance of plants to biological and abiotic stresses (Olanrewaju et al., 2019; Oppenheimer-Shaanan et al., 2022; Chepsergon and Moleleki, 2023). In addition, rhizosphere bacteria can also promote plant growth and development and absorb soil heavy metal pollution (Segura and Ramos, 2013; Rathi and Nandabalan, 2017; Ravelo-Ortega et al., 2023). Plants can in turn regulate microbial communities and recruit special functional microbial populations through root exudates (Adele et al., 2021; Xu et al., 2022). Rhizosphere bacteria can also respond to different environmental pressures by regulating microbial populations and functions and developing a series of response mechanisms (Amini Hajiabadi et al., 2021; Yin et al., 2021; Gao et al., 2024). Due to their rapid reproduction rate, rhizosphere bacteria have become an important ecological indicator reflecting the status of plant rhizosphere soil (Rossmann et al., 2020; Xiao et al., 2021; Song et al., 2022). The mine is also a good source for separating polluted bioremediation strains and plant growth-promoting strains (Ma et al., 2019; Amini Hajiabadi et al., 2021; Li et al., 2022a; Yang et al., 2022). Previous studies have found that the diversity of rhizosphere bacteria is closely related to the health status of plants (Zhang et al., 2021; Wu et al., 2023). However, the impact of kaolin mining on the diversity and function of rhizosphere bacteria in surrounding plants, as well as the response strategies of rhizosphere bacteria, is currently unclear.

In this study, we used 16S rRNA high-throughput sequencing to study the effects of kaolin mining on the rhizosphere bacterial communities and functions of three local plants (*Conyza bonariensis*, *Artemisia annua*, and *Dodonaea viscosa*) and analyzed the response strategies of rhizosphere bacterial communities to kaolin mining for the first time. The research results fill the gap in the ecological effects of kaolin mining, remind us to pay attention to the environmental risks of kaolin mining, and provide ideas for the ecological restoration of kaolin mining pollution.

## Materials and methods

### Rhizosphere soil collection and DNA extraction

Sampling of rhizosphere soil was conducted in the vicinity of Panzhihua city, Sichuan, China, in an area of kaolin mining from three native plants: *Conyza bonariensis*, *Artemisia annua*, and *Dodonaea viscosa*. The soils in the rhizosphere of *Conyza bonariensis*, *Artemisia annua*, and *Dodonaea viscosa*, sourced from a kaolin mining area, were denoted KL-Cbo, KL-Aan, and KL-Dvi, respectively. Three types of rhizosphere soils from non-kaolin mining areas with similar environmental conditions were used as control samples, namely, CK-Cbo, CK-Aan, and CK-Dvi. For use as a blank control soil sample, bulk soil taken from non-kaolin mining areas in similar environments was designated as a control. Each sample had three biological replicates. The rhizosphere soils were obtained by extracting the plant roots and shaking off the soil adhered to the exterior of the roots, which were then placed in self-sealing bags. For bacterial diversity analysis, 50 grams of soil was gathered from each sample. Subsequently, the 21 samples were transferred to the laboratory in an ice bag for DNA extraction and 16S rRNA sequencing. A soil DNA kit (D5625-02, OMEGA, CA, USA) was used to extract total genomic DNA from the soil samples. The quality and concentration of the extracted DNA were evaluated by running it on 1% (w/v) agarose gels and using Qubit 4.0 (Thermo Fisher, Massachusetts, USA).

### PCR amplification and detection

To achieve a concentration of 1 ng/μL for the extracted genomic DNA, sterile water was used, and the 16S rRNA V3-V4 regions were amplified via primers (341F: 5′-CCTACGGGAGGCAGCAG-3′; 806R: 5′-GGACTACNVGGGTWTCTAAT-3′) that included a barcode. The PCRs were conducted using T100 Thermal Cycler (Bio-Rad, CA, USA) with 15 microliters of Phusion® High-Fidelity PCR Master Mix from New England Biolabs, 2 microliters of forward and reverse primers, and 10 nanograms of template DNA. The initial step of the thermal cycling process entailed denaturing at 98°C for 1 min, repeating this process 30 times with 10 s of denaturing, 30 s of annealing at 50°C, 30 s of elongation at 72°C, and finally concluding with preservation at 72°C for 5 min (Li Q. et al., 2024; Xiang et al., 2024). Subsequent to the PCR, the products were blended with an equal volume of loading buffer containing SYBR green, and the combination was analyzed by electrophoresis on a 2% (w/v) agarose gel to detect the PCR products. After PCR amplification, the PCR products were purified using the Qiagen Gel Extraction Kit (Qiagen, Germany).

### Library preparation, sequencing, and raw data processing

Following the manufacturer’s instructions, sequencing libraries were created using the TruSeq® DNA PCR-Free Sample Preparation Kit (Illumina, USA), and index codes were added as part of the process. To assess the quality of the libraries, the Qubit@ 2.0 Fluorometer (Thermo Scientific) and the Agilent Bioanalyzer 2100 system were both utilized. The library was sequenced with the Illumina NovaSeq 6000 platform, producing 250-bp paired-end reads. Identification of the reads was based on their barcodes, with the barcode and primer sequences subsequently removed. FLASH V1.2.7 (Magoc and Salzberg, 2011) was used to merge paired-end reads. To guarantee the highest quality of tags, raw tags were filtered in line with the quality control process of QIIME 2 as part of the quality control procedure (Caporaso et al., 2010). The tags were compared to the Silva reference database (v.138.1) to identify any chimeric sequences, which were then eliminated from the dataset (Quast et al., 2013).

### ASV cluster and species annotation

For each ASV, a representative sequence was identified to facilitate annotation. The Silva database v.138.1 was used together to assign taxonomic details to each representative sequence (Quast et al., 2013). To investigate the phylogenetic relationship between the ASVs and the alterations in the most abundant species in distinct samples or groups, MUSCLE v3.8.3 was utilized to carry out multiple sequence alignments (Edgar, 2004). A sequence number benchmark was adopted to standardize the abundance information of ASVs, in line with the sample with the least number of sequences. The normalized data were subsequently utilized for further analysis of alpha and beta diversity.

### Alpha and beta diversity analyses

An analysis of species diversity was conducted for each sample using seven indices, namely, Observed species, Chao1, Shannon, Simpson, Pielou evenness, Faith_pd, and Good’s coverage. For the calculation of these indices, QIIME 2 was used, and the results were then displayed with R 2.15.3 (Caporaso et al., 2010). To assess the diversity of a community, three indices were chosen: the number of species observed, the Chao1 estimator, and Pielou evenness. To ascertain the diversity of a community, the Shannon index and Simpson index were used. A beta diversity analysis was implemented to measure the variation in species complexity between samples. A non-metric multidimensional scaling (NMDS) analysis and principal coordinate analysis (PCoA) were conducted using the R vegan software package.

### Functional prediction of rhizosphere bacteria

By using Phylogenetic Investigation of Communities by Reconstruction of Unobserved States 2 (PICRUSt2) (Douglas et al., 2018, 2020), we can make an informed inference about the function of rhizosphere bacteria based on our projections of gene ontology (GO), KO, and pathway databases (Kanehisa et al., 2004; Mistry et al., 2021). To reduce the dimension of the original variables, cluster analysis was preceded by the application of PCoA using the FactoMineR and ggplot2 packages in R v2.15.3.

### Soil physical and chemical property testing

We tested the physical and chemical properties of the obtained soil (Li et al., 2022b). Soil pH was measured using a pH meter. The loss-on-ignition method was used to estimate soil organic matter content. The Kjeldahl method was used for determining available nitrogen in soils. Available phosphorus in soils was detected using the Murphy–Riley colorimetric method. Flame photometry is used for the determination of available potassium in soil extract. X-ray fluorescence (XRF) spectroscopy was used to analyze the SiO_2_ content. Inductively coupled plasma optical emission spectrometry (ICP-OES) was used to detect elements in soils, including aluminum, cadmium (Cd), and chromium (Cr). The atomic absorption spectrometry (AAS) method was used for the determination of copper in soil extracts.

### Statistical analysis

To assess the magnitude of the disparities between the samples, we conducted a statistical analysis using R v2.15.3. The *t*-test was used to compare two sets of samples, while Tukey’s test was used for more than two sets. A *p*-value of less than 0.05 was considered indicative of a statistically significant divergence between various groups.

## Results

### Quality control and processing of sequencing data

Utilizing 16S rRNA high-throughput sequencing, we examined the rhizosphere bacteria associated with *C. bonariensis*, *A. annua*, and *D. viscosa* in a kaolin mining area and a non-mining area in the vicinity. As shown in Supplementary Figure S1, the rarefaction curves of rhizosphere bacterial ASVs in different samples demonstrate that with the increase in sequencing reads, the number of observed species gradually increased, and the curves flattened after the sequencing reads exceeded 20,000, indicating the adequacy of the sequencing reads to reflect the overall community structure of rhizosphere bacteria. Following the removal of chimeras, low-quality, and short sequences, the average number of clean reads per sample for downstream analysis was 59,405. These clean reads were then clustered into ASVs at a similarity threshold of 97%.

### Alpha diversity indices

**Figure 1:**
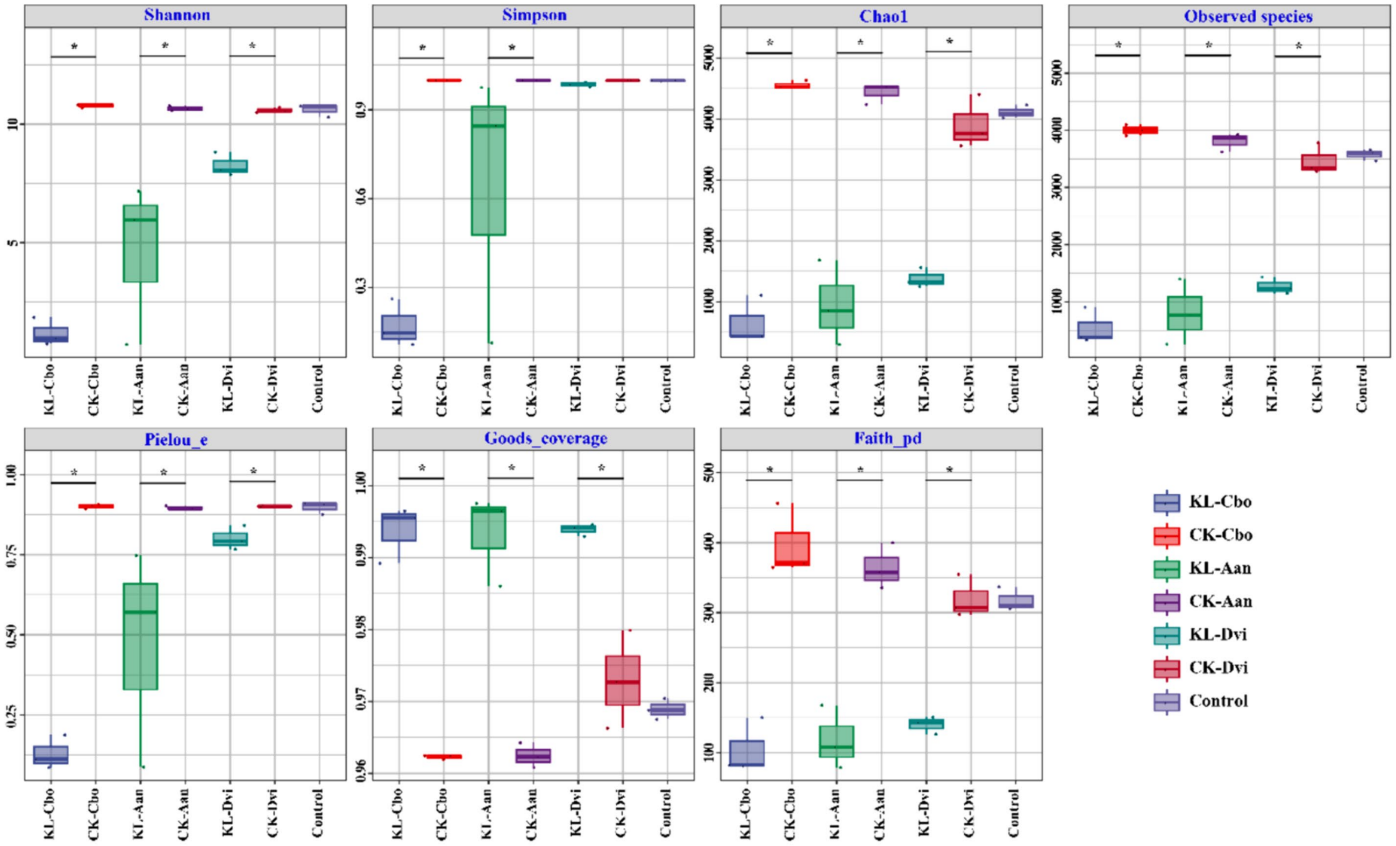
Box plots of rhizosphere bacterial diversity and richness in different samples. Asterisk indicates a significant difference between the two samples at the *p* < 0.05 level. KL-Cbo, *Conyza bonariensis* rhizosphere soil from kaolin mining area; KL-Aan, *Artemisia annua* rhizosphere soil from kaolin mining area; KL-Dvi, *Dodonaea viscosa* rhizosphere soil from kaolin mining area; CK-Cbo, *Conyza bonariensis* rhizosphere soil from non-kaolin mining area; CK-Aan, *Artemisia annua* rhizosphere soil from non-kaolin mining area; CK-Dvi, *Dodonaea viscosa* rhizosphere soil from non-kaolin mining area; control, bulk soil from non-kaolin mining area.

Analysis of species diversity and richness was conducted using seven indices, including Shannon, Simpson, Chao1, observed species, Pielou evenness, Good’s coverage, and Faith_pd (Figure 1). The results showed that the bulk soil samples and three plant rhizosphere soil samples from non-mining areas had similar bacterial diversity, as indicated by the Shannon and Simpson indices. However, kaolin mining significantly decreased the Shannon index of the three local plants and the Simpson index of *C. bonariensis* and *A. annua* rhizosphere soils from kaolin mining areas (*p* < 0.05). Additionally, the Chao1, observed species, and Pielou evenness indices of the rhizosphere soils of these three local plants were significantly reduced in kaolin mining areas compared to rhizosphere soil samples from non-kaolin mining areas (*p* < 0.05). Conversely, the Good’s coverage and Faith_pd indices of the rhizosphere soils of these three local plants significantly increased in kaolin mining areas (*p* < 0.05).

### Taxonomic analyses of bacterial communities

**Figure 2:**
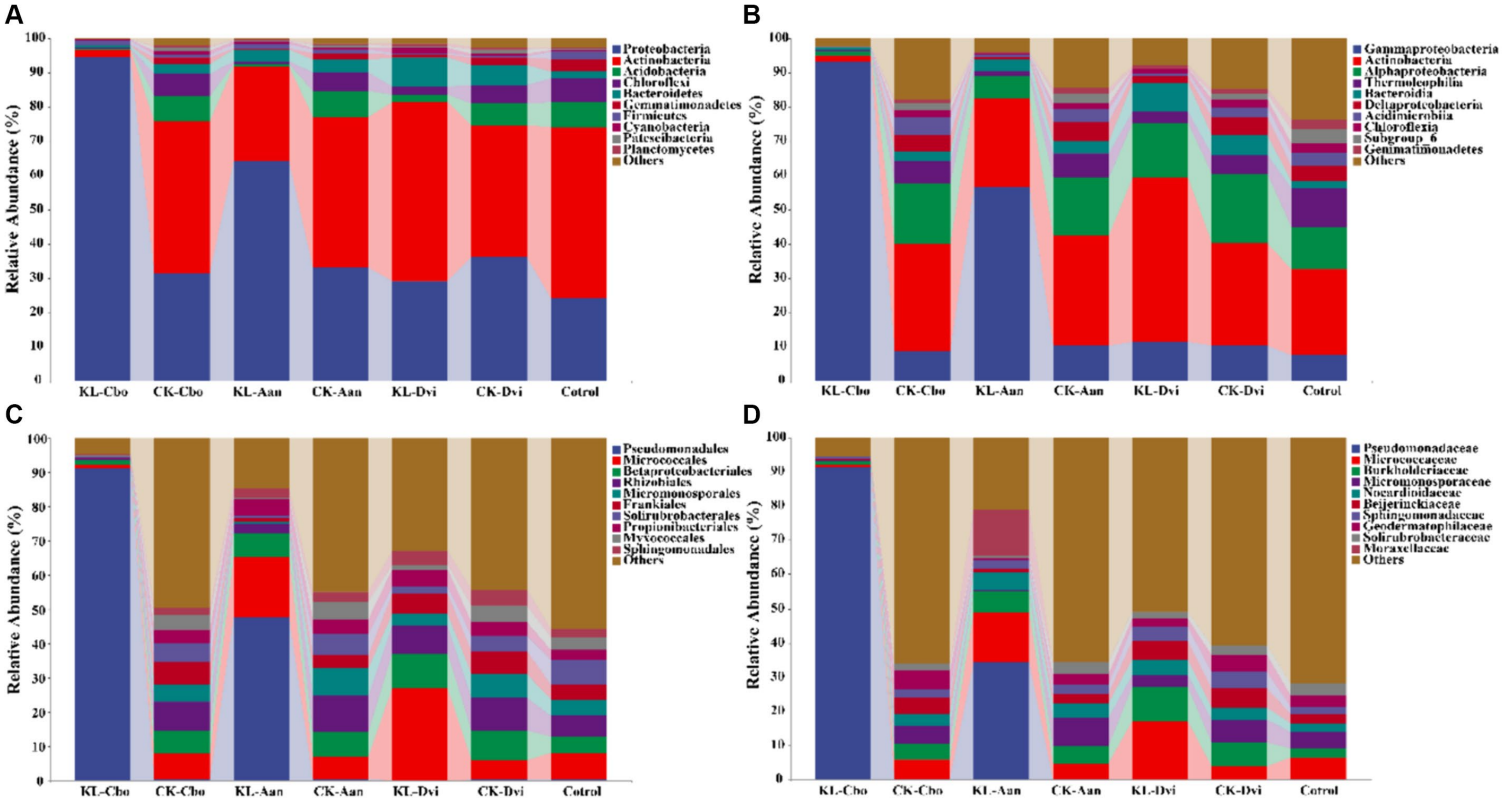
Cumulative bar charts of relative abundance of taxa (top 10) at the phylum **(A)**, class **(B)**, order **(C)**, and family **(D)** levels. KL-Cbo, *Conyza bonariensis* rhizosphere soil from kaolin mining area; KL-Aan, *Artemisia annua* rhizosphere soil from kaolin mining area; KL-Dvi, *Dodonaea viscosa* rhizosphere soil from kaolin mining area; CK-Cbo, *Conyza bonariensis* rhizosphere soil from non-kaolin mining area; CK-Aan, *Artemisia annua* rhizosphere soil from non-kaolin mining area; CK-Dvi, *Dodonaea viscosa* rhizosphere soil from non-kaolin mining area; control, bulk soil from non-kaolin mining area.

The abundance of the 10 most abundant phyla in different samples was compared (Figure 2A). Proteobacteria was the most abundant phylum in all samples, accounting for 44.65%, followed by Actinobacteria (36.97%), Acidobacteria (4.52%), and Chloroflexi (3.93%). The bulk soil sample had the lowest Proteobacteria abundance (average 24.16%), while the abundance of Proteobacteria in rhizosphere soil increased significantly (*p <* 0.05). The KL-Cbo sample had the highest Proteobacteria abundance, and the KL-Cvi sample had the highest Acidobacteria abundance (average 52.25%). Kaolin mining caused a significant increase in Proteobacteria abundance in the KL-Cbo and KL-Aan samples and a significant decrease in the KL-Dvi sample compared to the corresponding control sample from the non-mining area (*p <* 0.05).

At the class level, Gammaproteobacteria was the most abundant class in all samples, with an average of 28.27%, followed by Actinobacteria (27.65%), Alphaproteobacteria (13.05%) and Thermoleophilia (5.05%) (Figure 2B). Gammaproteobacteria abundance was significantly higher in rhizosphere soil samples than in bulk soil samples (*p <* 0.05). Moreover, the abundance of Gammaproteobacteria in the KL-Cbo and KL-Aan samples was significantly higher than that in the CK-Cbo and CK-Aan samples (*p <* 0.05). Actinobacteria abundance in the rhizosphere soil of *C. bonariensis* and *A. annua* planted in the kaolin mining area was lower than that in the non-kaolin mining area, while Actinobacteria was enriched in *D. viscosa* rhizosphere soil from the kaolin mining area compared to the non-kaolin mining area (*p <* 0.05).

At the order level, Pseudomonadales was the most abundant order in all samples, with an average of 20.08%, followed by Micrococcales (10.40%), Betaproteobacteriales (6.57%), and Rhizobiales (6.54%) (Figure 2C). Kaolin mining had an effect on the abundance of Pseudomonadales in *C. bonariensis* and *A. annua* rhizosphere soils, with an increase relative to samples from non-mining areas (*p <* 0.05). Micrococcales abundance was significantly decreased in *C. bonariensis* and significantly increased in the other two plant rhizosphere soils from the kaolin mining area compared to the corresponding control samples from the non-mining area (*p <* 0.05). Additionally, kaolin mining significantly decreased the abundance of Betaproteobacteriales in *C. bonariensis* rhizosphere soil (*p <* 0.05).

At the family level, Pseudomonadaceae (18.08%), Micrococcaceae (7.52%), Burkholderiaceae (5.26%), and Micromonosporaceae (4.13%) had the highest average abundance in all samples (Figure 2D). Kaolin mining was associated with an increase in the abundance of Pseudomonadaceae in the rhizosphere soils of *C. bonariensis* and *A. annua*, compared to the corresponding soil samples from the non-mining area (*p <* 0.05). Additionally, the abundance of Micrococcaceae was found to be higher in the rhizosphere soils of *A. annua* and *D. viscosa* from the kaolin mining area, while it decreased in the rhizosphere soil of *C. bonariensis* (*p <* 0.05). Burkholderiaceae showed a decrease in the rhizosphere soil of *C. bonariensis*, while an increase was observed in *A. annua* and *D. viscosa* from the kaolin mining area compared to the corresponding rhizosphere soils from the non-kaolin mining area (*p <* 0.05).

**Figure 3:**
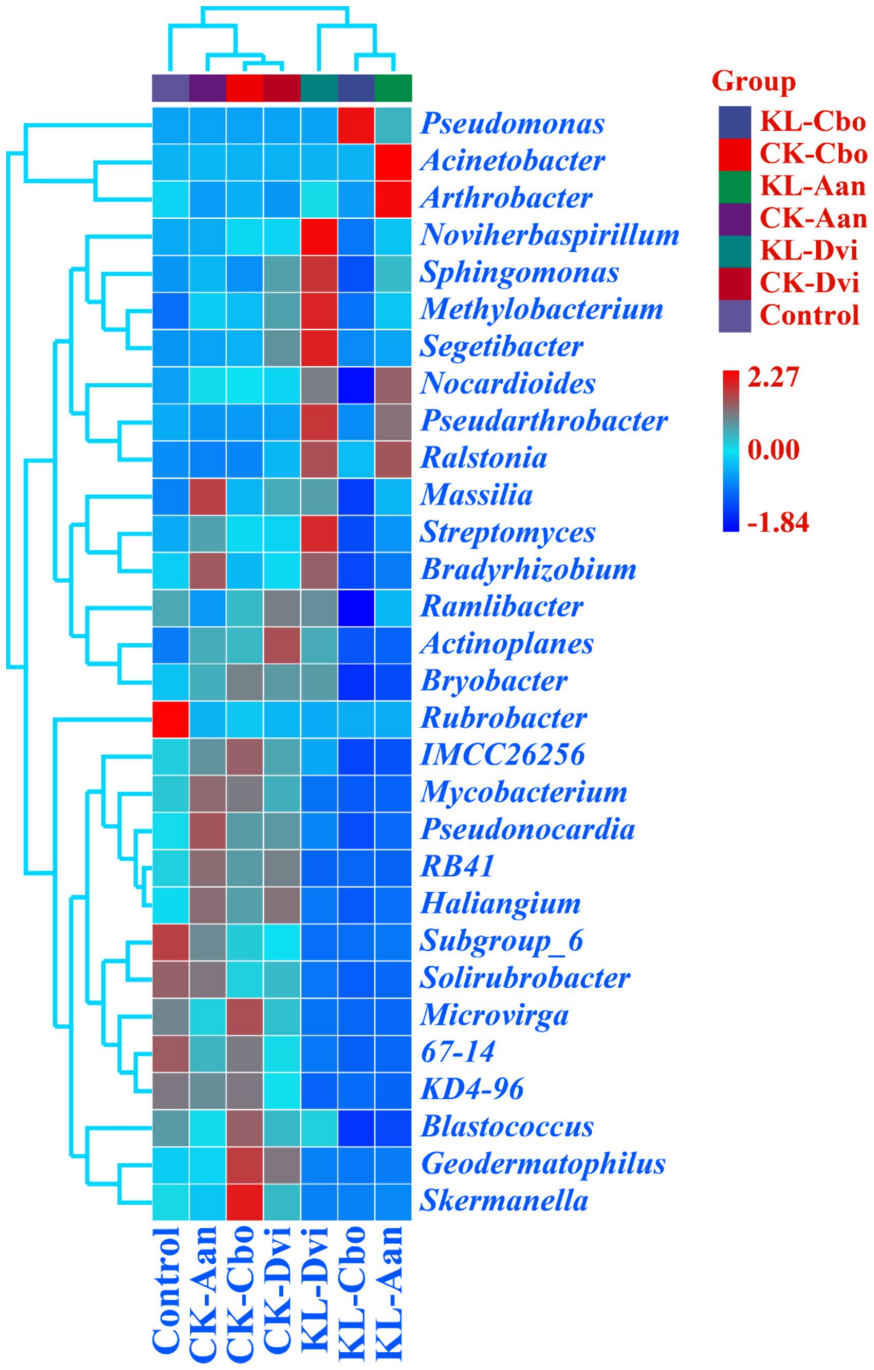
Heat map analysis of the 30 genera with the highest abundance in different samples. The relative abundance of different genera is represented by different color blocks, with colors ranging from blue to red indicating an increase in the relative abundance of the genera. KL-Cbo, *Conyza bonariensis* rhizosphere soil from kaolin mining area; KL-Aan, *Artemisia annua* rhizosphere soil from kaolin mining area; KL-Dvi, *Dodonaea viscosa* rhizosphere soil from kaolin mining area; CK-Cbo, *Conyza bonariensis* rhizosphere soil from non-kaolin mining area; CK-Aan, *Artemisia annua* rhizosphere soil from non-kaolin mining area; CK-Dvi, *Dodonaea viscosa* rhizosphere soil from non-kaolin mining area; control, bulk soil from non-kaolin mining area.

At the genus level, *Pseudomonas* was the most abundant genus in all samples, accounting for 18.03% (Figure 3). This was followed by *Nocardioides* (2.15%), Acinetobacter (1.96%), *Pseudarthrobacter* (1.92%), *Sphingomonas* (1.77%), and *Streptomyces* (1.70%). Significantly, the abundance of *Pseudomonas* was higher in the rhizosphere samples of *C. bonariensis* and *A. annua* from the kaolin mining area compared to the corresponding control sample (*p <* 0.05). Additionally, the abundance of *Nocardioides*, *Pseudarthrobacter*, and *Sphingomonas* decreased in *C. bonariensis* and increased in the other two rhizosphere soils from the mining area compared to the corresponding control samples (*p <* 0.05). Furthermore, the abundance of *Streptomyces* decreased in *C. bonariensis* and *A. annua* and increased in *D. viscosa* rhizosphere soils in the kaolin mining area relative to the corresponding rhizosphere soils in the non-kaolin mining area (*p <* 0.05). Moreover, the influence of kaolin mining was found to reduce the abundance of *Blastococcus*, *Actinoplanes*, *Solirubrobacter*, *RB41*, *Mycobacterium*, *Geodermatophilus*, and *Skermanella* compared to the corresponding control samples from non-kaolin mining areas in the rhizosphere soil of three local plants (*p <* 0.05). In contrast, *Ralstonia* was enriched in the rhizosphere soil of three local plants from the kaolin mining area compared to the rhizosphere soil of non-kaolin mining areas (*p <* 0.05). In addition, we noticed that *Actinoplanes*, *RB41*, and *Mycobacterium* were enriched in the rhizosphere of the three plants compared to bulk soils, while kaolin mining significantly reduced their abundance (*p <* 0.05).

### Structural differentiation of microbial communities

**Figure 4:**
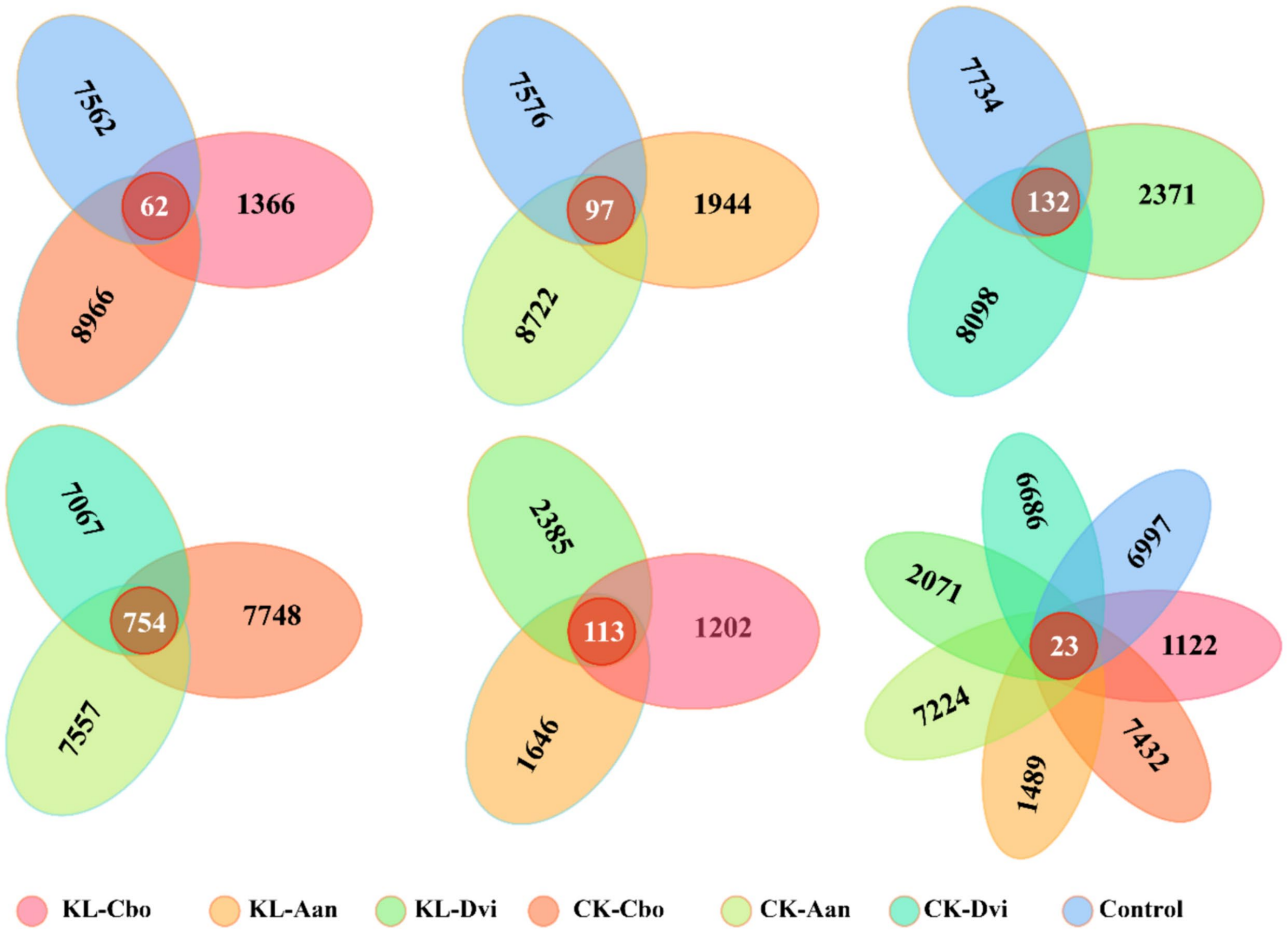
Shared and unique ASVs between different samples. KL-Cbo, *Conyza bonariensis* rhizosphere soil from kaolin mining area; KL-Aan, *Artemisia annua* rhizosphere soil from kaolin mining area; KL-Dvi, *Dodonaea viscosa* rhizosphere soil from kaolin mining area; CK-Cbo, *Conyza bonariensis* rhizosphere soil from non-kaolin mining area; CK-Aan, *Artemisia annua* rhizosphere soil from non-kaolin mining area; CK-Dvi, *Dodonaea viscosa* rhizosphere soil from non-kaolin mining area; control, bulk soil from non-kaolin mining area.

Analysis of specific and shared ASVs between different samples revealed that the rhizosphere bacterial communities of *C. bonariensis*, *A. annua*, and *D. viscosa* in the non-kaolin mining area were distinct from the control samples, with 8,966, 8,722, and 8,098 specific ASVs, respectively (Figure 4). In the rhizosphere soil of the kaolin mining area, *C. bonariensis*, *A. annua*, and *D. viscosa* demonstrated 1,366, 1944, and 2,371 specific ASVs, respectively, when compared to the non-kaolin mining area. Moreover, when comparing the rhizosphere bacteria of different host species in the non-kaolin mining area, *C. bonariensis*, *A. annua*, and *D. viscosa* displayed 7,748, 7,557, and 7,067 specific ASVs, respectively. Additionally, the CK-Cbo, CK-Aan, and CK-Dvi samples contained 754 shared ASVs. In terms of rhizosphere bacteria of different host species planted in the kaolin mining area, *C. bonariensis*, *A. annua*, and *D. viscosa* exhibited 1,202, 1,646, and 2,385 specific ASVs, respectively, along with 113 common ASVs. Ultimately, all samples contained 23 core ASVs, and each sample contained 1,122–7,432 specific ASVs.

**Figure 5:**
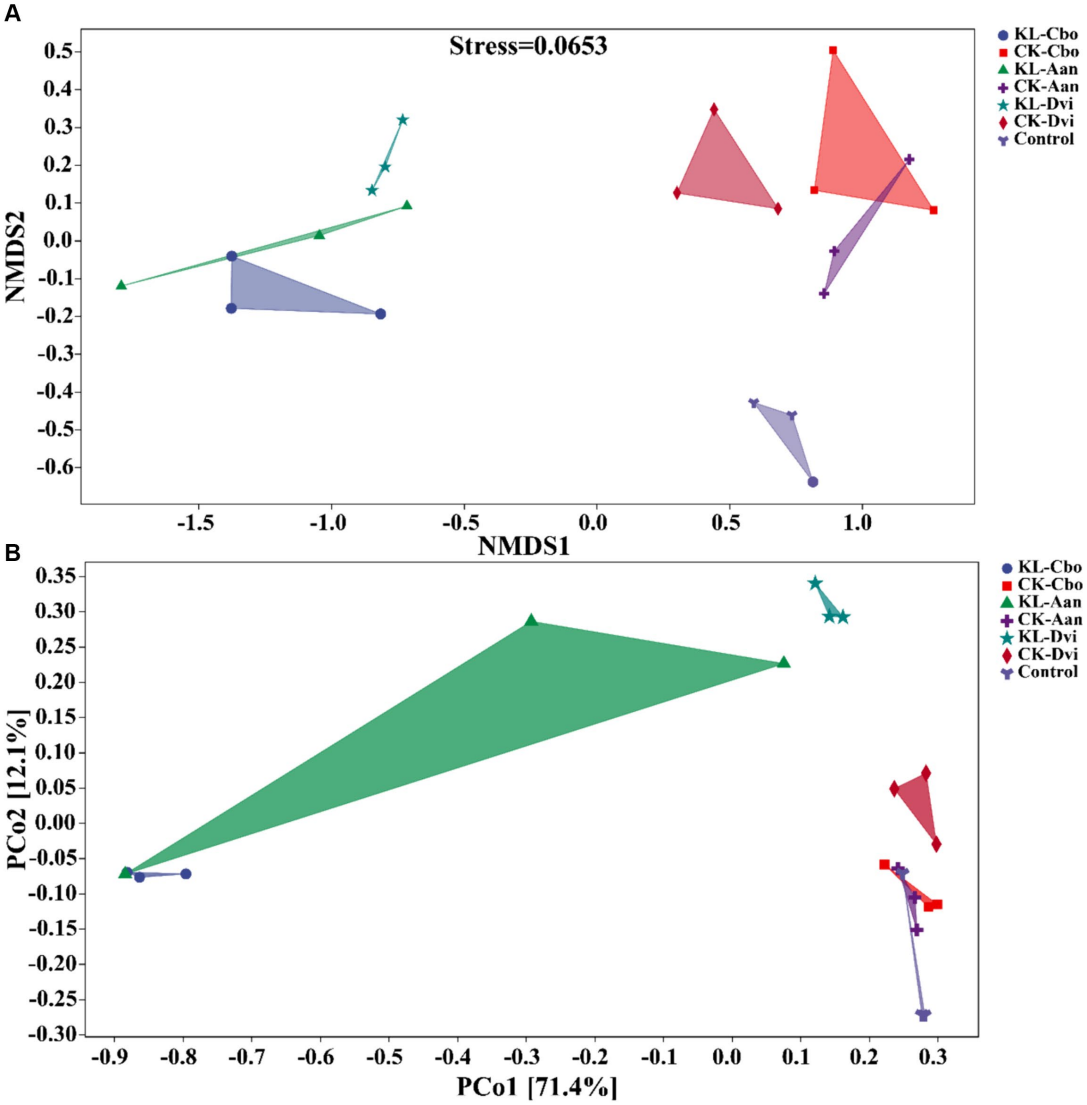
Beta diversity analysis of rhizosphere bacterial communities in different samples based on NMDS **(A)** and PCoA analysis **(B)**. KL-Cbo, *Conyza bonariensis* rhizosphere soil from kaolin mining area; KL-Aan, *Artemisia annua* rhizosphere soil from kaolin mining area; KL-Dvi, *Dodonaea viscosa* rhizosphere soil from kaolin mining area; CK-Cbo, *Conyza bonariensis* rhizosphere soil from non-kaolin mining area; CK-Aan, *Artemisia annua* rhizosphere soil from non-kaolin mining area; CK-Dvi, *Dodonaea viscosa* rhizosphere soil from non-kaolin mining area; control, bulk soil from non-kaolin mining area.

Our analysis of the variations in bacterial communities among different samples, using NMDS and PCoA (Figure 5), revealed that the rhizosphere bacterial community structure of the three local plants was distinct from that of the CK sample, and the community similarity between the CK-Cbo sample and CK sample was higher than that of the other two samples. Kaolin mining was also found to have a significant effect on the rhizosphere soil bacterial community structure compared to non-kaolin mining. The NMDS and PCoA analyses showed that kaolin mining had a greater influence on the rhizosphere bacterial community structure variation than genotype (species), indicating the reshaping effect of kaolin mining on the rhizosphere bacterial community of local plants.

### Function prediction of the bacterial community

Approximately 85.14% of the bacterial genes were classified into three categories according to the COG database: cellular processes and signaling, information storage and processing, and metabolism (Supplementary Figure S2A). Metabolism was the most enriched of these functions, accounting for 45.84% of the genes. The KO database classified all the bacterial genes into six categories, with metabolism being the most enriched at 82.83%, followed by genetic information processing (10.19%) and cellular processes (4.10%) (Supplementary Figure S2B). The bacterial genes can be classified into seven categories, namely, biosynthesis, degradation/utilization/assimilation, detoxification, generation of precursor metabolites and energy, glycan pathways, macromolecule modification, and metabolic clusters (Supplementary Figure S2C). Of these, biosynthesis was the most abundant, accounting for 62.40% of all samples, followed by degradation/utilization/assimilation (17.93%) and generation of precursor metabolites and energy (15.79%).

**Figure 6:**
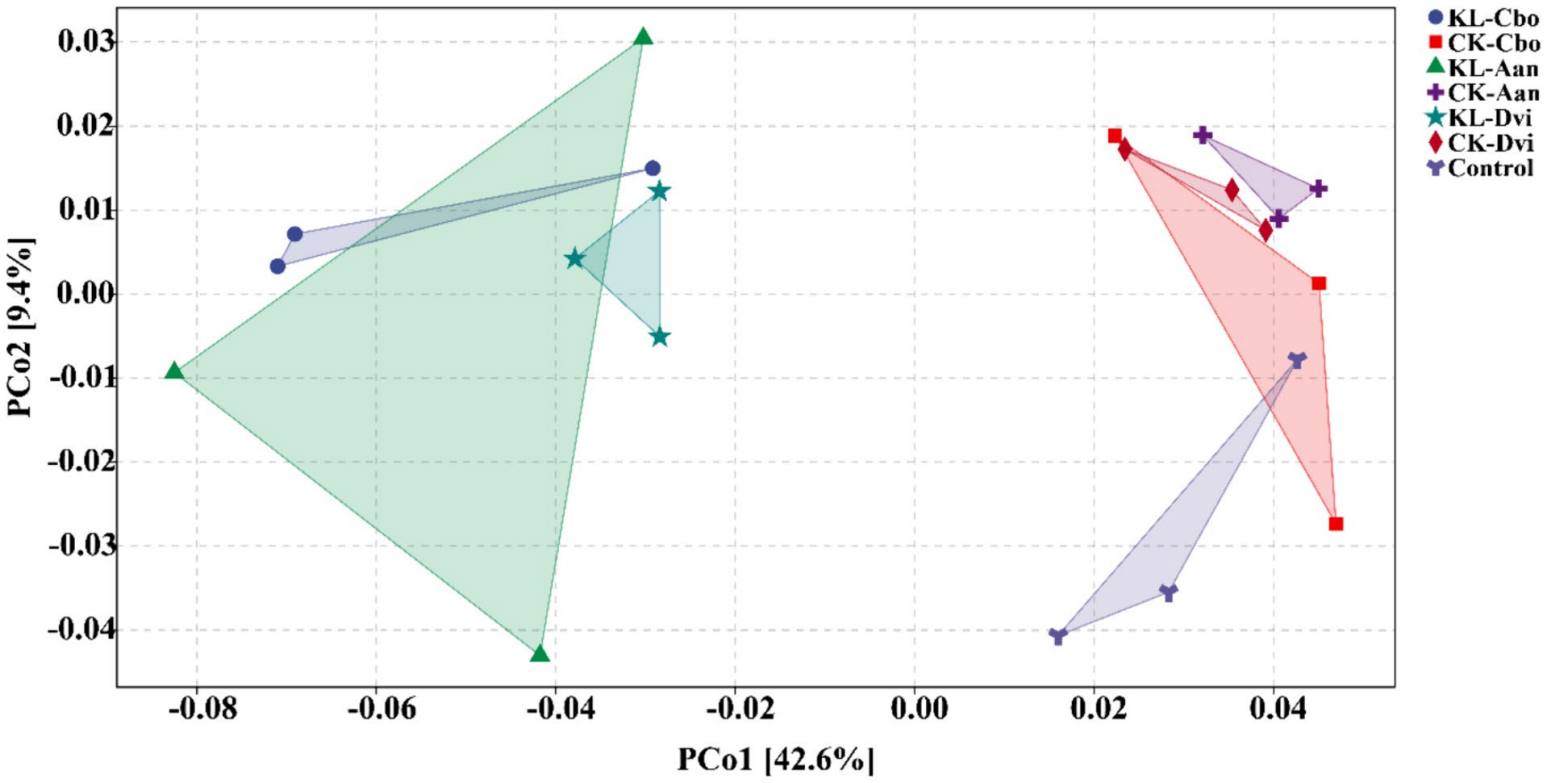
PCoA analysis of rhizosphere bacterial functions predicted by PICRUSt2. KL-Cbo, *Conyza bonariensis* rhizosphere soil from kaolin mining area; KL-Aan, *Artemisia annua* rhizosphere soil from kaolin mining area; KL-Dvi, *Dodonaea viscosa* rhizosphere soil from kaolin mining area; CK-Cbo, *Conyza bonariensis* rhizosphere soil from non-kaolin mining area; CK-Aan, *Artemisia annua* rhizosphere soil from non-kaolin mining area; CK-Dvi, *Dodonaea viscosa* rhizosphere soil from non-kaolin mining area; control, bulk soil from non-kaolin mining area.

By using PCoA (Figure 6), the variations in bacterial functions among the samples were assessed, showing that the function of rhizosphere soil was distinct from that of the control sample to some extent and that kaolin mining also resulted in a differentiation of bacterial functions in the rhizosphere of different local plants compared to samples from non-kaolin mining areas.

### Functional enrichment of rhizosphere bacteria

The rhizospheres of *C. bonariensis*, *A. annua*, and *D. viscosa* from non-mining areas had increased abundances of pyoverdine/dityrosine biosynthesis protein Dit1 (COG3207), serine protease inhibitor ecotin (COG4574), chromosome condensin MukBEF (COG3095), cell division protein DamX (COG3266), predicted nucleotidyltransferase (COG4849), uncharacterized protein (COG4861), and predicted nucleic acid-binding protein (COG5595) functions, respectively, compared to bulk soil samples (Supplementary Figure S3). Notably, COG3207, COG4849, and COG4861 were enriched in the rhizospheres of all three plants (*p <* 0.05). Conversely, the abundance of uncharacterized protein YkuJ (COG4703), sensor histidine kinase (COG3275), uncharacterized protein YuzB (COG4844), and uncharacterized protein YunC (COG3377) decreased in the rhizospheres of the three plants compared to bulk soil samples (*p <* 0.05).

In comparison with bulk soil samples, the rhizosphere of *C. bonariensis* from non-mining areas was enriched with the functions of glycerol-3-phosphate cytidylyltransferase (ko00980), while bile secretion (ko04976) and the cell receptor signaling pathway (ko04622) were reduced (Supplementary Figure S4). *A. annua*, on the other hand, increased systemic lupus erythematosus (ko05322) and melanin-concentrating hormone receptor 1 (ko04320), while dTMP kinase (ko00943) and ko04622 decreased (*p <* 0.05). For *D. viscosa*, the functions of DNA-directed RNA polymerase subunit alpha (ko03040), ko04320, ko05322, calpain-12 (ko04740), and G protein-coupled receptor 83 (ko04210) were enriched in its rhizosphere soil in comparison with the bulk sample, and ko04622 was reduced in both the rhizospheres of *C. bonariensis* and *A. annua* relative to the bulk soil sample (*p <* 0.05).

In comparison to bulk soil samples, the rhizospheres of *C. bonariensis*, *A. annua*, and *D. viscosa* from non-mining areas were enriched with the pathways phospholipases (LIPASYN-PWY), 2-propanediol degradation (PWY-7013), isoprene biosynthesis II (PWY-7391), mevalonate pathway II (PWY-6174), starch degradation III (PWY-6731), sucrose biosynthesis III (PWY-7347), heparin degradation (PWY-7644), sucrose biosynthesis I (SUCSYN-PWY), and fosfomycin biosynthesis (PWY-5757), and PWY-6174, PWY-6731, PWY-7391, PWY-622, and LIPASYN-PWY, respectively (Supplementary Figure S5) (*p <* 0.05). Notably, the pathways LIPASYN-PWY, PWY-7347, and SUCSYN-PWY were enriched in the rhizosphere soil of all three plants from non-mining areas (*p <* 0.05). Conversely, the functions of benzoyl-CoA degradation II (CENTBENZCOA-PWY), methanogenesis from H_2_ and CO_2_ (METHANOGENESIS-PWY), L-methionine salvage cycle I (PWY-7528), thiazole biosynthesis II (PWY-6891), and the superpathway of thiamin diphosphate biosynthesis II (PWY-6895) were found to be decreased in abundance in the rhizosphere soil of these plants compared to bulk soil samples (*p <* 0.05).

### Functional enrichment of rhizosphere bacteria from a kaolin mining area

**Figure 7:**
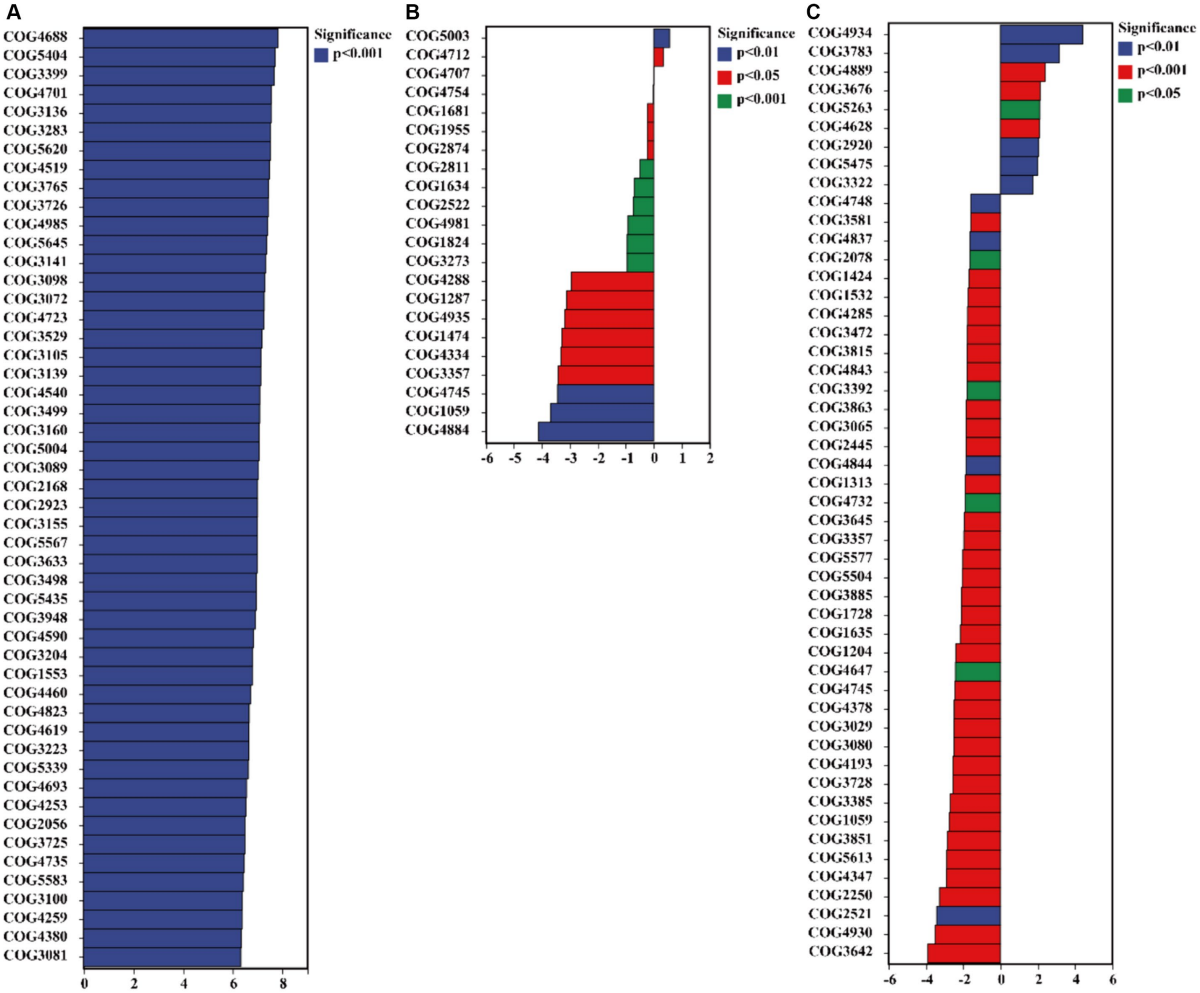
Significant differentially expressed functions of bacteria in the rhizosphere of plants from kaolin mining areas and non-kaolin mining areas based on the COG database. The vertical axis represents the ID of COG, the horizontal axis represents the value of log2 (fold change), and different colors indicate significant differences between samples at different levels. **(A)** KL-Cbo vs. CK-Cbo; **(B)** KL-Aan vs. CK-Aan; **(C)** KL-Dvi vs. CK-Dvi.

The results of the COG database indicate that *C. bonariensis* rhizosphere soil from kaolin mining areas has an increased presence of the GlpM family (COG3136), uncharacterized protein (COG4701), uncharacterized protein (COG3399), cell division inhibitor SulA (COG5404), and uncharacterized protein (COG4688) compared to the control samples (*p <* 0.05) (Figure 7). Furthermore, uncharacterized protein (COG4712) and Mu-like prophage protein gp37 (COG5003) were enriched in *A. annua* rhizosphere soil from kaolin mining areas compared to non-kaolin mining areas (*p <* 0.05). Additionally, *D. viscosa* rhizosphere soils from kaolin mining areas showed enrichment of glucan-binding domain (COG5263), transposase and inactivated derivatives (COG3676), predicted helicase (COG4889), soluble cytochrome b562 (COG3783), and serine protease, subtilase family (COG4934) compared to non-kaolin mining areas (*p <* 0.05). Conversely, the functionality of uncharacterized protein (COG4288) and predicted nucleotide-binding protein (COG3581) was decreased in *A. annua* and *D. viscosa* rhizosphere soil samples from kaolin mining areas compared to the control samples (*p <* 0.05).

**Figure 8:**
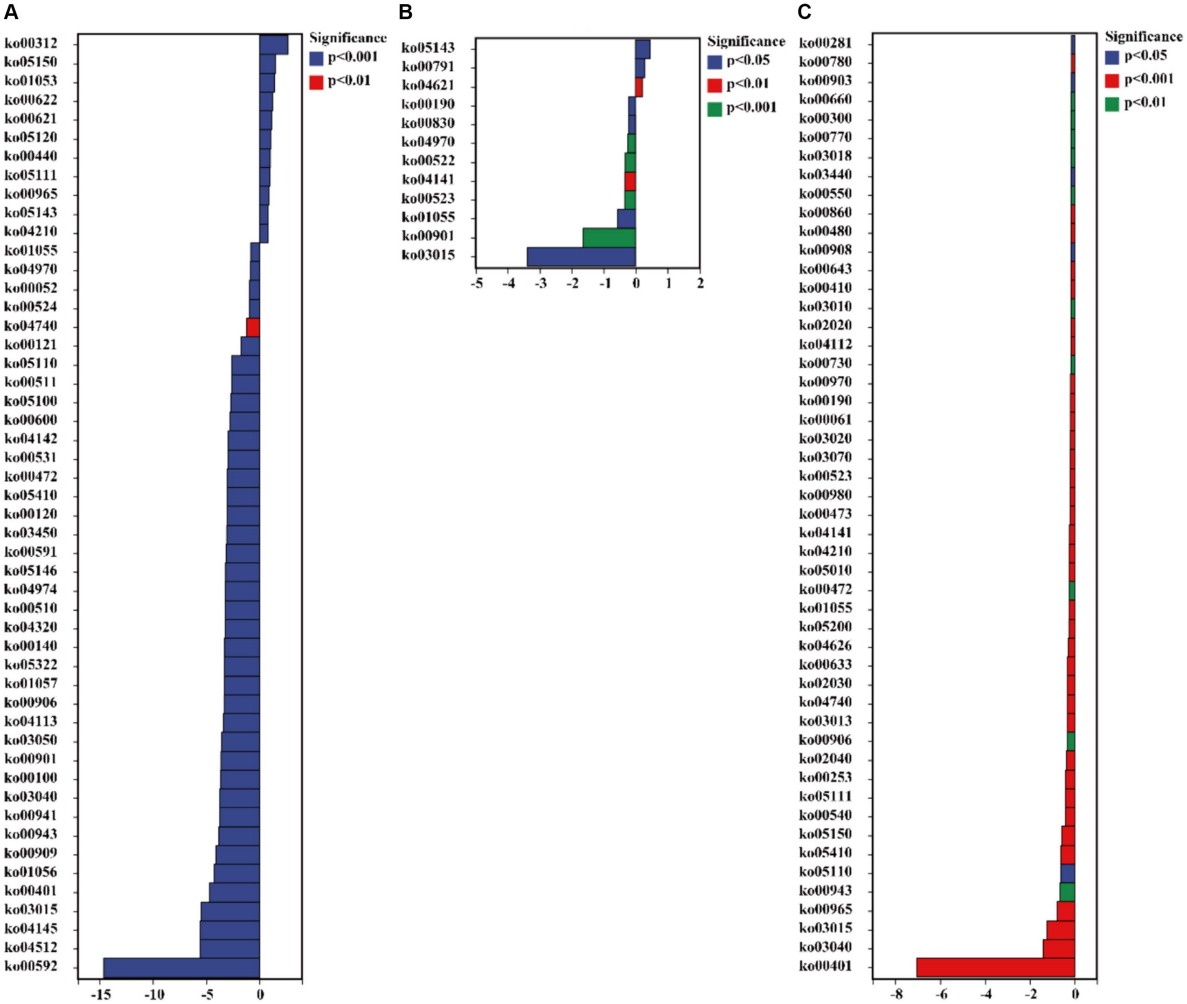
Significant differentially expressed functions of bacteria in the rhizosphere of plants from kaolin mining areas and non-kaolin mining areas based on the KO database. The vertical axis represents the ID of KO, the horizontal axis represents the value of log2 (fold change), and different colors indicate significant differences between samples at different levels. **(A)** KL-Cbo vs. CK-Cbo; **(B)** KL-Aan vs. CK-Aan; **(C)** KL-Dvi vs. CK-Dvi.

The functionalities of glucosamine-phosphate N-acetyltransferase (ko00621), arylamine N-acetyltransferase (ko00622), gluconolactonase (ko01053), tumor necrosis factor receptor superfamily member 13B (ko05150), and wingless-type MMTV integration site family (ko00312) in *C. bonariensis* rhizosphere soil were significantly increased due to kaolin mining (*p <* 0.05) (Figure 8). In contrast, G protein-coupled receptor family C group 5 member D (ko04621), tRNA dimethylallyltransferase (ko00791), and tumor necrosis factor receptor superfamily member 6B (ko05143) were enriched in *A. annua* rhizosphere soil from the kaolin mining area compared to the non-kaolin mining area (*p <* 0.05). On the other hand, calpain-12 (ko04740), adrenergic receptor beta-1 (ko04141), and methyl-coenzyme M reductase beta subunit (ko00401) were decreased in *C. bonariensis*, *A. annua*, and *D. viscosa* rhizosphere soil samples from the kaolin mining area compared to the control samples, respectively (*p <* 0.05).

**Figure 9:**
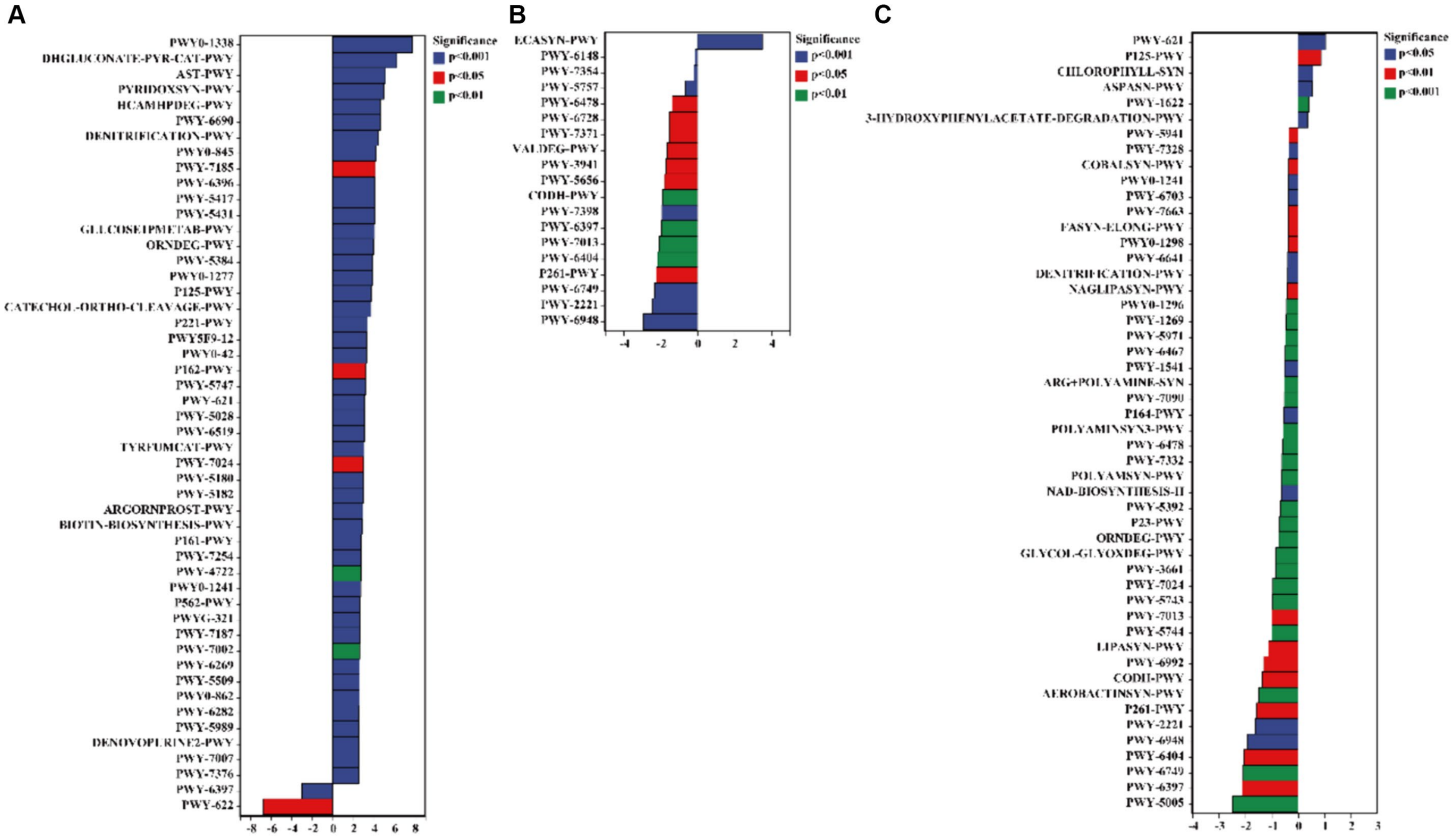
Significant differentially expressed functions of bacteria in the rhizosphere of plants from kaolin mining areas and non-kaolin mining areas based on the Pathway database. The vertical axis represents the ID of the pathway, the horizontal axis represents the value of log2 (fold change), and different colors indicate significant differences between samples at different levels. **(A)** KL-Cbo vs. CK-Cbo; **(B)** KL-Aan vs. CK-Aan; **(C)** KL-Dvi vs. CK-Dvi.

The results of the pathway analysis indicated that the functions of 3-phenylpropanoate (HCAMHPDEG-PWY), pyridoxal 5′-phosphate biosynthesis I (PYRIDOXSYN-PWY), L-arginine degradation II (AST-PWY), glucose degradation (DHGLUCONATE-PYR-CAT-PWY), and polymyxin resistance (PWY0-1338) were enhanced in the rhizosphere soil of *C. bonariensis* from the kaolin mining area compared to the control samples (*p <* 0.05) (Figure 9). Additionally, enterobacterial common antigen biosynthesis (ECASYN-PWY) was enriched in the rhizosphere soil of *A. annua* from the kaolin mining area compared to the non-kaolin mining area (*p <* 0.05). Furthermore, formaldehyde assimilation I (PWY-1622), the superpathway of L-aspartate and L-asparagine biosynthesis (ASPASN-PWY), chlorophyllide a biosynthesis I (CHLOROPHYLL-SYN), the superpathway of (R,R)-butanediol biosynthesis (P125-PWY), and sucrose degradation III (PWY-621) were enriched in the rhizosphere soil of *D. viscosa* from the kaolin mining area compared to that from the non-kaolin mining area (*p <* 0.05). Conversely, the functionality of mycolyl-arabinogalactan-peptidoglycan complex biosynthesis (PWY-6397), GDP-D-glycero-alpha (PWY-6478), and glycogen degradation II (PWY-5941) was reduced in the rhizosphere soil of *C. bonariensis*, *A. annua*, and *D. viscosa* from the kaolin mining area, respectively (*p <* 0.05).

### Correlation analysis of bacterial communities

**Figure 10:**
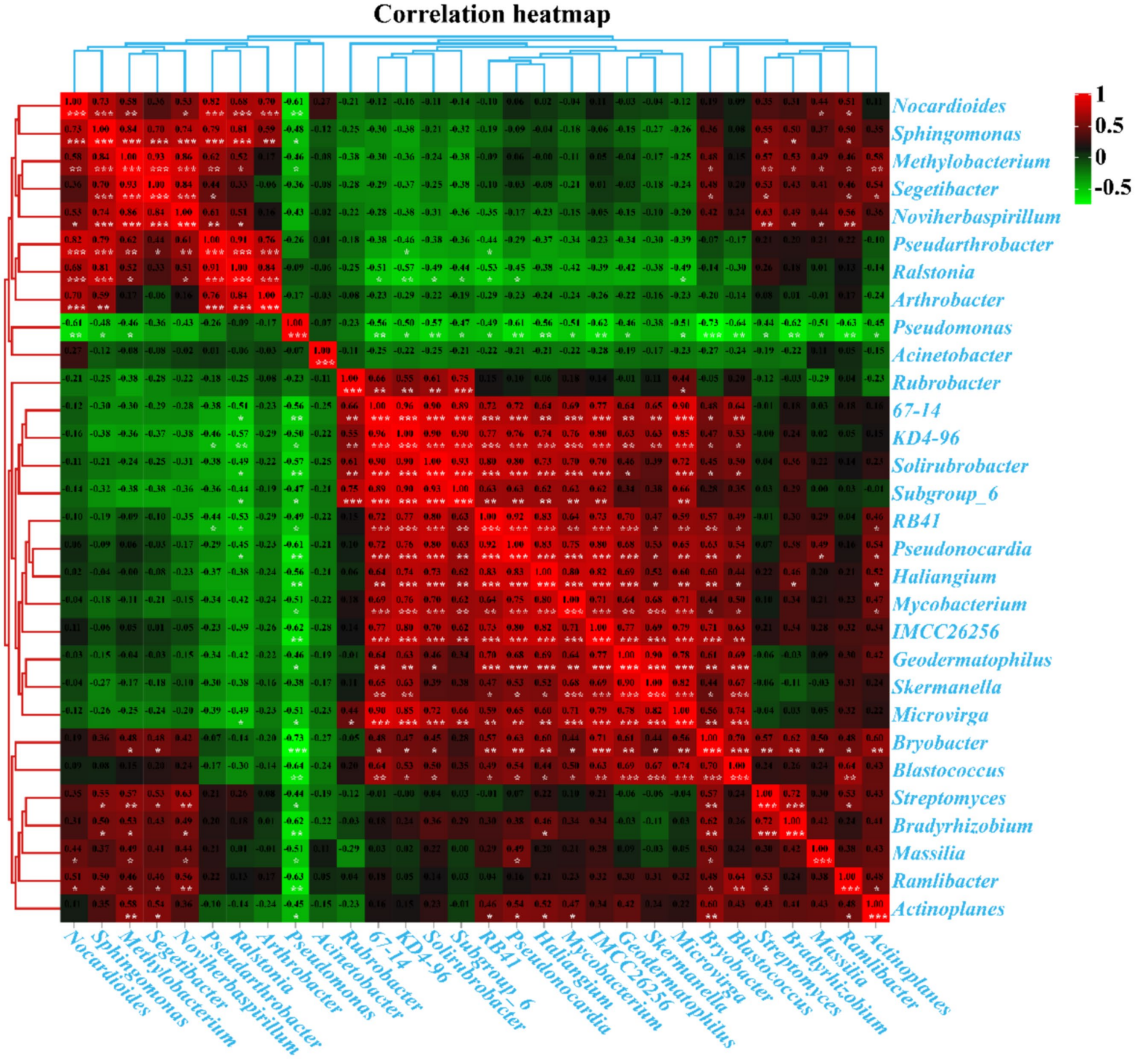
Pearson’s correlation analysis between the 30 genera with the highest abundance in different samples. Pearson’s correlation index is marked in a square color block, with a green color block indicating a negative correlation between two bacterial genera and a red color block indicating a positive correlation. One asterisk indicates a significant correlation between two bacterial genera at the *p* < 0.05 level, while two asterisks indicate a significant correlation between two bacterial genera at the *p* < 0.01 level, and three asterisks indicate a significant correlation between two bacterial genera at the *p* < 0.001 level.

Pearson’s correlation analysis revealed significant interactions between the top 30 genera with the highest abundance in kaolin mining areas (Figure 10). Positive correlations were observed between *Segetibacter*, *Methylobacterium*, *Pseudarthrobacter*, *Noviherbaspirillum*, *Sphingomonas*, and *Nocardioides*; *Haliangium*, *Pseudonocardia*, *RB41*, *Skermanella*, *Geodermatophilus*, *Mycobacterium*, KD4-96, 67–14, *Microvirga*, IMCC26256, *Solirubrobacter*, Subgroup_6, *Blastococcus*, and *Bryobacter* (*p <* 0.05). Additionally, *Bradyrhizobium* and *Streptomyces* were also found to be positively correlated (*p <* 0.05). On the other hand, negative correlations were observed between *Pseudonocardia*, *RB41*, KD4-96, 67–14, *Solirubrobacter*, Subgroup_6, and *Ralstonia* (*p <* 0.05). Similarly, *Haliangium*, *Pseudonocardia*, *Bradyrhizobium*, Streptomyces, *Geodermatophilus*, *Mycobacterium*, Massilia, *Microvirga*, IMCC26256, Ramlibacter, *Actinoplanes*, *Blastococcus*, *Bryobacter*, and *Pseudomonas* were found to be negatively correlated (*p <* 0.05). Moreover, *Methylobacterium*, *Sphingomonas*, *Nocardioides*, and *Pseudomonas* were also found to be negatively correlated (*p <* 0.05), suggesting a strong interaction between the dominant bacterial populations that collectively shape the composition and distribution of rhizosphere bacterial populations in kaolin mining areas.

### Determination of soil physical and chemical properties

We have further tested the physical and chemical properties of the collected soil (Supplementary Table S1). The results showed that kaolin mining led to soil acidification. Compared with the control, the content of silica and alumina in kaolin soil has significantly increased. In addition, the content of some heavy metals, including Cd, Cr, and Cu, has also been found to exceed the standard in kaolin mining areas. In addition, we further analyzed the correlation between soil physical and chemical properties and microbial indicators and found that the excessive heavy metal elements in soil were significantly positively correlated with the decrease of soil Shannon index (*p* < 0.05). In addition, the increase in heavy metal concentration led to a significant decrease (*p* < 0.05) in the abundance of *Blastococcus*, *Actinoplanes*, and *Solirubrobacter*, and a significant increase in the abundance of *Ralstonia* (*p* < 0.05).

## Discussion

Our study found that kaolin mining significantly reduced the diversity and community richness indicators of rhizosphere bacteria in three local plants, including the Shannon, Simpson, Chao1, observed species, and Pielou evenness indices. The results have demonstrated the negative effects of kaolin mining on the rhizosphere microecology of surrounding plants, which may be caused by the diffusion of heavy metals and other harmful components brought about by kaolin mining, as well as the deterioration of the physicochemical properties of plant rhizosphere soil (Wang et al., 2006; Roach et al., 2009). Previous studies have found that mining activities can have significant negative effects on the microbial diversity of surrounding soil, mainly due to the pollution leakage caused by mining activities, which is consistent with the results of this study (Zhao et al., 2020; Gallego et al., 2021; Qiu et al., 2024; Xiao et al., 2024; Xiong et al., 2024). Rhizosphere bacteria play an important role in plant growth and development, nutrient absorption, and tolerance to biotic and abiotic stresses (Alami et al., 2023; Chepsergon and Moleleki, 2023). Previous studies have shown that the diversity of rhizosphere bacteria in plants is closely related to their health and quality (Edwards et al., 2015; Stassen et al., 2021; Korenblum et al., 2022). The rich diversity of plant rhizosphere bacteria helps plants survive in adverse environments and cope with environmental pressures (Chapelle et al., 2016; Dessaux et al., 2016; Venturi and Keel, 2016). This study observed for the first time that kaolin mining had negative effects on local plant rhizosphere bacteria, reminding us to pay attention to the ecological protection of kaolin mining areas and take measures to protect local biological communities.

Further study has found that kaolin mining has caused the differentiation of rhizosphere bacterial communities of three local plants, and kaolin mining has become the main driving force for reshaping the rhizosphere bacterial communities of three local plants, indicating that the ecological effects of kaolin mining cannot be ignored. In addition, we also found that the abundance of *Blastococcus*, *Actinoplanes*, *Solirubrobacter*, *RB41*, *Mycobacterium*, *Geodermatophilus*, and *Skermanella* significantly decreased in the rhizosphere of three local plants from kaolin mining areas compared to soil samples from non-mining areas. *Blastococcus*, *Skermanella*, and *Geodermatophilus* are often found in deserts (Zhang et al., 2015; Subhash and Lee, 2016; Yang et al., 2019), *Actinoplanes* and *Solirubrobacter* are often found to be rooted in the plant rhizosphere (Luo et al., 2021; Yuan et al., 2023), and *RB41* is enriched in phosphate mining areas (Yang et al., 2022). The results indicate that these strains are relatively sensitive to the environmental pressure caused by kaolin mining. In addition, we noticed that *Actinoplanes*, *RB41*, and *Mycobacterium* were enriched in the rhizosphere of the three plants compared to bulk soils, while kaolin mining significantly reduced their abundance, indicating that kaolin mining affected the recruitment process of rhizosphere bacteria by local plants (Vives-Peris et al., 2020; Li Z. et al., 2022; Fan et al., 2023). Interestingly, we found that *Ralstonia* was enriched in the rhizosphere of three local plants from kaolin mining areas, indicating that it has good environmental tolerance and ecological effects. *Ralstonia* has been found to have good environmental adaptability, including tolerance to various heavy metals (Nies, 2000; Huang et al., 2021). Mineral mining areas are considered effective places for obtaining bioremediation bacterial resources, as they have good environmental adaptability and remediation potential (Mkandawire, 2013; McCarthy et al., 2017; Hao et al., 2021; Anupong et al., 2022). These bacterial resources can be further developed and utilized for bioremediation in mining areas (Cornu et al., 2017; Aguilar et al., 2020; Shylla et al., 2021). In addition, we also found that some bacteria were differentially enriched in the rhizosphere of three local plants in the kaolin mining area, indicating the shaping and screening of microbial communities by host plants. During the long-term coevolution and coadaptation process with host plants, rhizosphere bacteria have formed a series of mutually beneficial mechanisms (Zhalnina et al., 2018; Poudel et al., 2019; Prekrasna et al., 2022). The host plant creates a unique growth environment for rhizosphere bacteria through root secretion, thereby altering the composition of the rhizosphere bacterial community (Liu et al., 2019; Zeng et al., 2022; Lopes et al., 2023). Overall, we observed that kaolin mining affected the recruitment process of plant rhizosphere bacteria, becoming an important force driving the reshaping of the local plant rhizosphere bacterial community structure. During this process, we discovered several dominant bacterial populations that became an important resource for bioremediation in kaolin mining areas.

We further utilized PICRUSt2 to predict the function of rhizosphere bacterial communities based on three databases and found that kaolin mining also drove the functional differentiation of rhizosphere bacterial communities (Douglas et al., 2020). Compared to bulk soil, the three local plants each had different enriched community functions. The mining of kaolin further affected the functions of three local plant rhizosphere bacteria, some of which were the GlpM family (COG3136), glucan-binding domain (COG5263), transpose and inactivated derivatives (COG3676), glucose phase N-acetyltransferase (ko00621), arylamine N-acetyltransferase (ko00622), 3-phenylpropanoate (HCAMHPDEG-PWY), and pyridoxal 5′-phase biosynthesis I (PYRIDOXSYN-PWY), L-arginine degradation II (AST-PWY), and glucose degradation (DHGLUCONATE-PYR-CAT-PWY) were enhanced in the rhizosphere of different local plants. In contrast, predicted nucleoside binding protein (COG3581), calpain-12 (ko04740), acute receiver beta-1 (ko04141), methyl-coenzyme M reduction beta subunit (ko00401), and mycolyl-arabinogalactan-peptidoglycan complex biosynthesis (PWY-6397) decreased in the mining area, which reveals the impact of kaolin mining on the functional differentiation of rhizosphere bacteria and the response mechanism of rhizosphere bacteria to kaolin mining and environmental stress (Senthil Kumar et al., 2023).

## Conclusion

This study explored the impacts of kaolin mining on the bacterial communities and functions of the rhizosphere of three local plant species, namely, *C. bonariensis*, *A. annua*, and *D. viscosa*. The results revealed that kaolin mining had a detrimental effect on the diversity and richness of the rhizosphere bacteria of the three local plants. Kaolin mining also impacted the recruitment of the rhizosphere bacteria of three local plants. Interestingly, *Ralstonia* was enriched in the rhizosphere soils of the three plants from kaolin mining areas, indicating its strong environmental tolerance and ecological role. Kaolin mining was found to reduce the abundance of *Blastococcus*, *Actinoplanes*, *Solirubrobacter*, *RB41*, *Mycobacterium*, *Geodermatophilus*, and *Skermanella* in rhizosphere soils of three local plants compared to the corresponding control samples from non-kaolin mining areas, which can be used as an ecological indicator for detecting soil health in kaolin mining areas. Kaolin mining caused a change in the functional diversity of the rhizosphere bacteria of the three local plants, each displaying a distinct set of functions to deal with the stress caused by mining activities. This study has improved our understanding of the microecological effects of kaolin mining and has provided a basis for creating effective soil microecological health monitoring indicators and strategies for soil pollution remediation in kaolin mining areas.

## Data availability statement

The original contributions presented in the study are publicly available. This data can be found at: NCBI SRA (https://www.ncbi.nlm.nih.gov/sra), accession numbers SRR29446543 and SRR29446544.

## Author contributions

WG: Data curation, Formal analysis, Writing – original draft. XC: Formal analysis, Writing – review & editing. JH: Formal analysis, Writing – review & editing, Data curation. AS: Formal analysis, Writing – review & editing. YR: Formal analysis, Visualization, Writing – review & editing. PW: Formal analysis, Funding acquisition, Writing – review & editing. QL: Formal analysis, Validation, Writing – review & editing.
