# Supplementary material for: The impact of kaolin mining activities on bacterial diversity and community structure in the rhizosphere soil of three local plants
Source: Front Microbiol. 2024 Sep 9;15:1424687. doi: 10.3389/fmicb.2024.1424687 (PMC11417686; doi:10.3389/fmicb.2024.1424687)
Supplement: Supplementary file 1 [file Data_Sheet_1.pdf]

Impact of Kaolin Mining Activities on the Bacterial Diversity and Community Structure in the Rhizospheric Soil of Three Local Plants

Running title: Rhizosphere bacteria affected by kaolin

Wei Gao[a], Xiaodie Chen[a], Jing He[a], Ajia Sha[a], Yuanhang Ren[a], Peng Wu[b]*, Qiang Li[a*]

a Clinical Medical College & Affiliated Hospital of Chengdu University, Key Laboratory of Coarse Cereal Processing, Ministry of Agriculture and Rural Affairs, Chengdu University, Chengdu, Sichuan, China;

b Yunnan Plateau Characteristic Agricultural Industry Research Institute, Yunnan Agricultural University, Kunming, Yunnan, China.

***Corresponding author:**

Peng Wu (382856897@qq.com) and Qiang Li (liqiang02@cdu.edu.cn)

Phone: +86-028-84616653;

***Present address: Chengdu University, Chengdu 610106, Sichuan, China**

Table S1 The physicochemical properties of rhizosphere soil from kaolin mining and non-kaolin mining areas.

Sample	pH	Organic matter (g/kg)	Available Phosphorus (mg/kg)	Available Potassium (mg/kg)	Available Nitrogen (mg/kg)	SiO_2 (%)	Al_2O_3 (%)	Cu (mg/kg)	Cr (mg/kg)	Cd (mg/kg)
CK-Cbo	7.13±0.05a	40.09±0.57ab	17.12±0.12a	143.69±1.51ab	167.32±1.39a	50.23±0.67b	11.03±0.13b	1.09±0.01c	11.87±0.12d	0.06±0.00d
KL-Cbo	5.67±0.03c	36.54±0.23b	15.61±0.10b	135.12±1.89c	152.13±1.44b	68.12±0.43a	27.69±0.29a	13.27±0.02a	67.89±0.33b	0.67±0.01bc
CK-Aan	6.67±0.02b	41.54±0.45a	16.18±0.09ab	145.12±1.72a	160.54±1.22a	50.21±0.25b	10.87±0.22b	1.22±0.01c	13.69±0.02cd	0.02±0.00d
KL-Aan	5.32±0.01c	34.12±0.66c	14.89±0.08c	133.44±1.11c	150.29±1.89b	69.28±0.66a	25.34±0.15a	12.35±0.02b	70.23±0.11b	0.58±0.01c
CK-Dvi	6.32±0.04bc	42.33±0.32a	15.39±0.09b	144.66±1.47a	148.66±1.21c	50.19±0.48b	10.22±0.14b	1.03±0.01c	14.12±0.02c	0.03±0.00d
KL-Dvi	5.11±0.02d	37.12±0.41b	14.29±0.12c	140.67±1.52bc	135.68±1.19c	69.67±0.56a	28.33±0.13a	14.60±0.02a	72.39±0.05ab	0.80±0.01a
Control	6.97±0.02a	40.32±0.32a	16.85±0.12a	145.67±1.53a	168.34±1.30a	50.15±0.42b	10.33±0.21b	1.08±0.02c	10.86±0.13d	0.04±0.00d

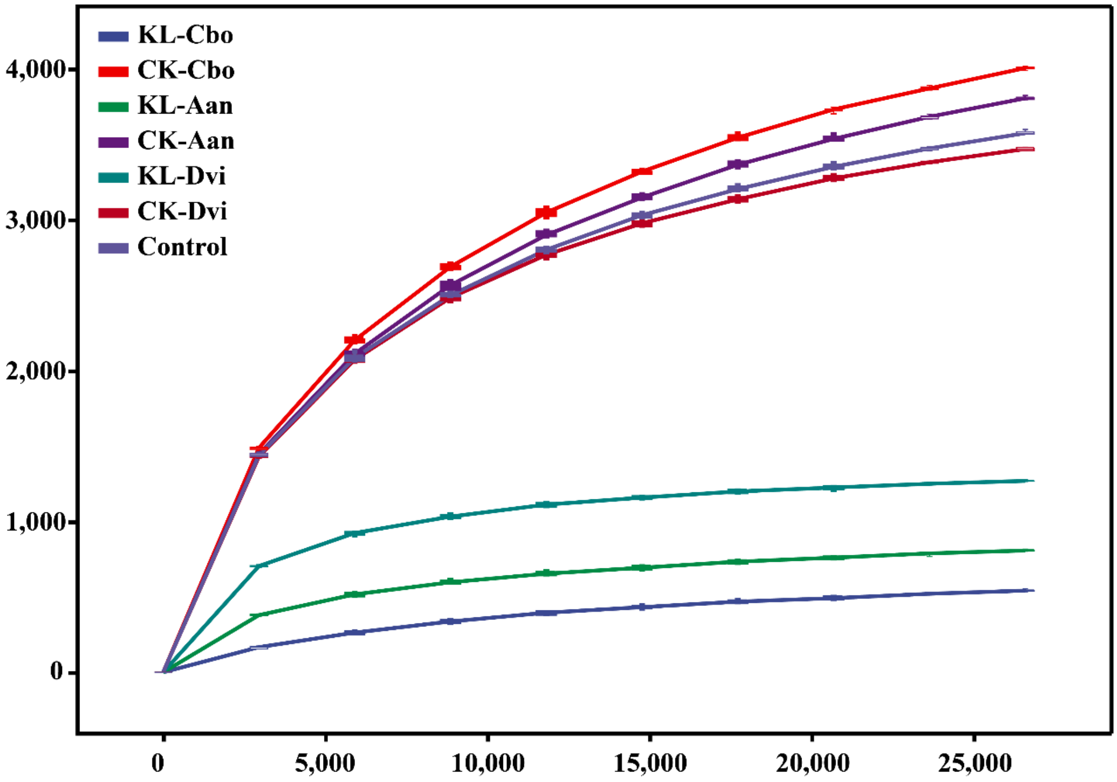

Figure S1 Rarefaction curves of rhizosphere bacterial ASVs in different samples. KL-Cbo, *Conyza bonariensis* rhizosphere soil from kaolin mining area; KL-Aan, *Artemisia annua* rhizosphere soil from kaolin mining area; KL-Dvi, *Dodonaea viscosa* rhizosphere soil from kaolin mining area; CK-Cbo, *Conyza bonariensis* rhizosphere soil from non-kaolin mining area; CK-Aan, *Artemisia annua* rhizosphere soil from non-kaolin mining area; CK-Dvi, *Dodonaea viscosa* rhizosphere soil from non-kaolin mining area; Control, bulk soil from non-kaolin mining area.

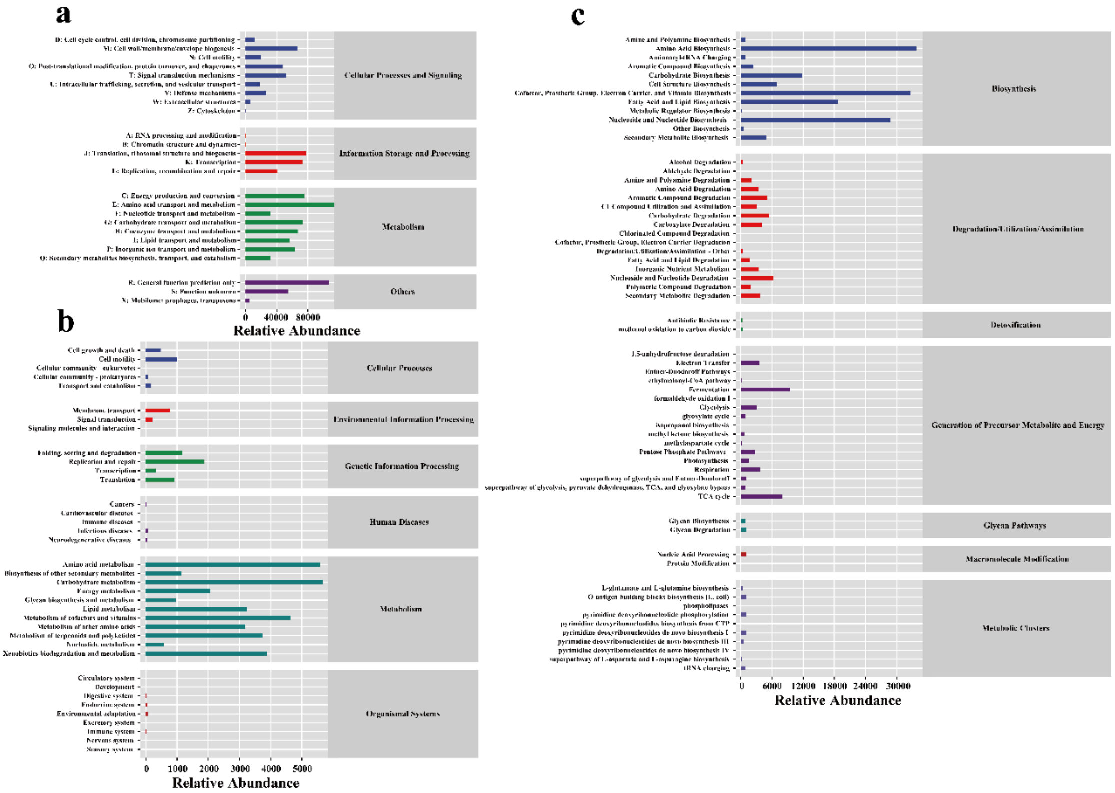

Figure S2 Functional enrichment analysis based on COG (a), KO (b), and pathway (c) databases predicted by PICRUSt2.

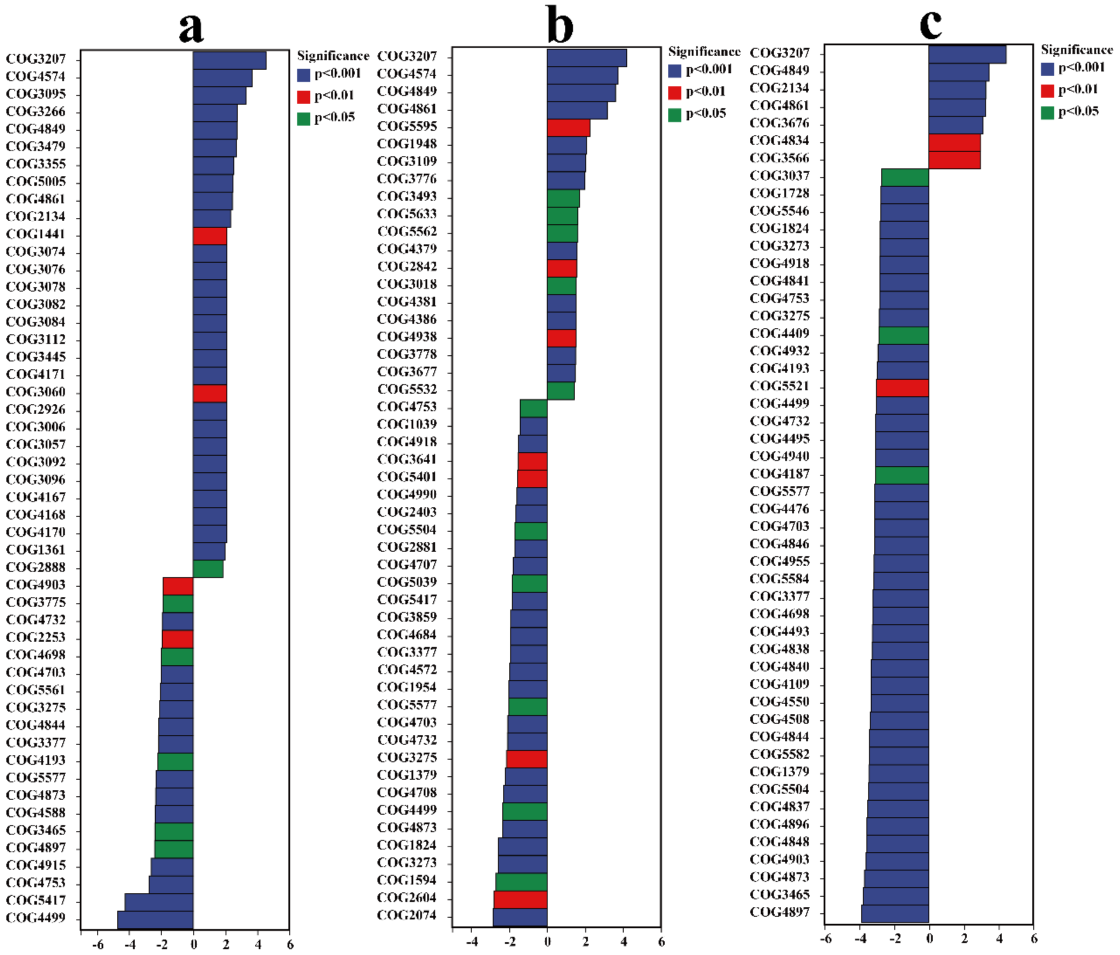

Figure S3 Significant differentially expressed functions of bacteria in the rhizosphere of plants from non-kaolin mining areas and bulk soil based on the COG database. The vertical axis represents the ID of COG, the horizontal axis represents the value of log2 (fold change), and different colors indicate significant differences between samples at different levels. a, CK-Cbo vs Control; b, CK-Aan vs Control; c, CK-Dvi vs Control.

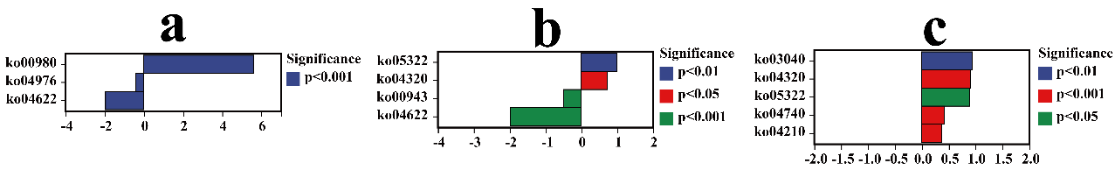

Figure S4 Significant differentially expressed functions of bacteria in the rhizosphere of plants from non-kaolin mining areas and bulk soil based on the KO database. The vertical axis represents the ID of KO, the horizontal axis represents the value of log2 (fold change), and different colors indicate significant differences between samples at different levels. a, CK-Cbo vs Control; b, CK-Aan vs Control; c, CK-Dvi vs Control.

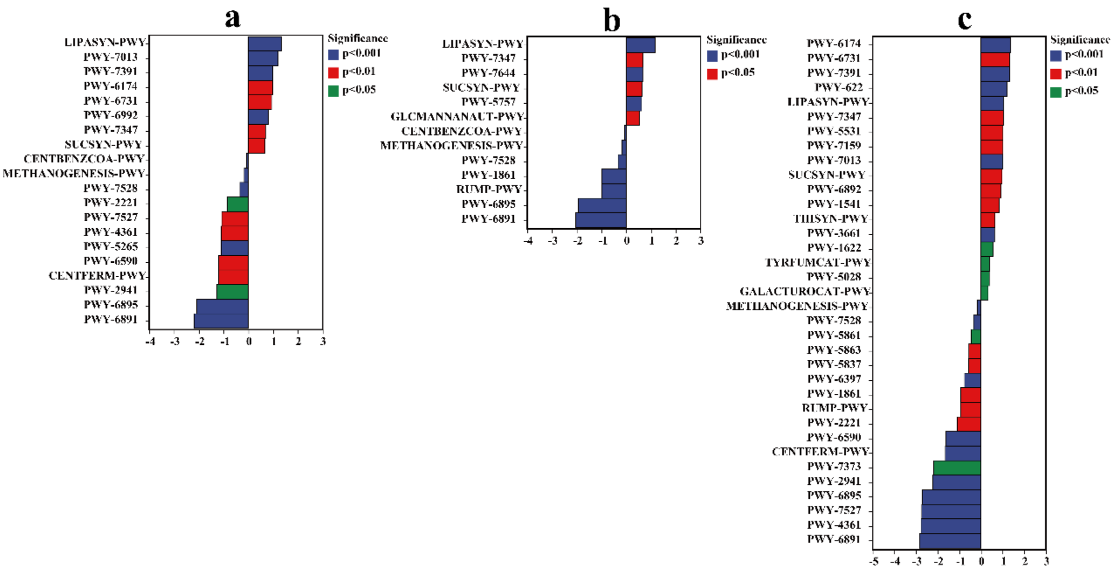

Figure S5 Significant differentially expressed functions of bacteria in the rhizosphere of plants from non-kaolin mining areas and bulk soil based on the Pathway database. The vertical axis represents the ID of Pathway, the horizontal axis represents the value of log2 (fold change), and different colors indicate significant differences between samples at different levels. a, CK-Cbo vs Control; b, CK-Aan vs Control; c, CK-Dvi vs Control.
